# Study on Fatigue Performance of Pulsed Tungsten Inert Gas Welding Joint of Duplex Stainless Steel Thin Tube

**DOI:** 10.3390/ma17010109

**Published:** 2023-12-25

**Authors:** Chaofei Yang, Wenjian Zheng, Renfu Wang, Jiajia Niu, Lei Sun, Mao Cheng, Xianjing Lu, Zhou Zhou, Jianguo Yang

**Affiliations:** 1 Luoyang Ship Material Research Institute, Luoyang 471000, China; 547321950@163.com (C.Y.); wangrenfu@725.com.cn (R.W.); niujiajia@725.com.cn (J.N.); sunlei@725.com.cn (L.S.); 2 College of Mechanical Engineering, Zhejiang University of Technology, Hangzhou 310014, China; zhouzhou1879@163.com (Z.Z.); yangjg@zjut.edu.cn (J.Y.); 3 Zhejiang Academy of Special Equipment Science, Hangzhou 310020, China; chengmao@163.con (M.C.); luxainjing@163.com (X.L.); 4 Key Laboratory of Special Equipment Safety Testing Technology of Zhejiang Province, Hangzhou 310020, China

**Keywords:** shielding gas nitriding, thin-walled pipe, butt joint, fatigue properties, P-TIG welding

## Abstract

To solve the shortage of austenite phase precipitation caused by nitrogen loss in the welding process of UNS S2205 duplex stainless steel (DSS), shielding gas nitriding was investigated by adding different N_2_ contents in Ar shielding gas during the welding process. A good thin-walled pipe butt joint was formed using the pulsed tungsten inert gas (P-TIG) welding method with Ar-N_2_ shielding gas. High cycle fatigue tests of the weld joints were conducted to study the effect of shielding gas nitriding on the fatigue properties. Fatigue tests at three stress levels of 225 MPa, 270 MPa, and 360 MPa were carried out on the weld joints with different N_2_ contents, and the fatigue samples were all fractured in the high temperature heat-affected zone (H-HAZ). Within the current process parameters, the fatigue life of the 4 vol.% N_2_ welded joints was optimal. Fatigue striations appeared in the fatigue crack propagation zone, and the transient fracture zone was similar to the tensile fracture. Under the low-stress level, the area of the crack propagation zone under 4 vol.% N_2_ was the highest, the tear ridges all expanded around the crack source area, and the fatigue crack propagation zone presented a radial distribution. The proliferation and expansion of dislocations were mainly carried out in the austenite grains, and the dislocation density of the fatigue specimens under 4 vol.% N_2_ was smaller than that of the Ar specimens. Shielding gas nitriding effectively improved the balance of the two-phase ratio and the hardness of austenite phase, optimized the internal slip system, inhibited the proliferation of dislocations in the austenite phase, and improved the fatigue life of weld joints.

## 1. Introduction

A large number of pipe welding operations are usually required due to long transportation distances and pipeline bifurcation in the chemical industry and the fields of oil and gas transmission and marine transportation [1,2,3]. Super long pipes and complicated curved pipe structures are common in engineering applications. Pipe welding technology can be used to complete pipeline construction at low cost and high efficiency according to corresponding engineering needs [4]. Traditional manual welding has difficulty producing the high-quality weld joints required for pipeline docking; thus, semi-automatic and automatic welding have gradually become mainstream in pipeline construction. With the development of pipeline construction in the direction of precision, lightweightness, and the diversification of application fields, high-quality and high-efficiency welding technology and various thin-walled pipe welding parts will be applied to practical projects [5,6,7].

Austenite and ferrite two-phase microstructure balance is needed in the weld joint of duplex stainless steel (DSS). In the welding process of DSS, 100 vol.% argon (Ar) or 100 vol.% carbon dioxide (CO_2_) is usually used as the welding shielding gas. With these shielding gases, the adoption of appropriate welding methods and welding heat input can improve the two-phase ratio [8,9,10,11]. However, to obtain a weld joint with two more balanced phases and higher mechanical properties, it is not enough to only regulate the welding heat input. Alcantara et al. [12] used Ar as the shielding gas to weld 2304 duplex stainless steel by TIG and found that the loss of N in the molten pool was serious. The ferrite content in both the weld metal and HAZ was relatively high compared to BM. Sun et al. [13] studied the microstructure and secondary phase precipitation of UNS S32101 duplex stainless steel flux-cored arc welding (FCAW) weld joints under 100% CO_2_ shielding gas and found that the proportion of austenite in the HAZ region was usually about 30 vol.%, which was much lower than that of the weld metal. According to the above research, it is found that the ferrite content in the HAZ of duplex stainless steel weld joints using 100 vol.% Ar or 100 vol.% CO_2_ as the welding shielding gas is generally high [12,13]. The reason for the high ferrite content in the HAZ is that all duplex stainless steels solidify in the ferritic form and subsequently change to the austenitic structure during solid-state phase transformation. During solid-state cooling of the weld pool, the transformation of ferrite to austenite is driven by austenite nucleation at ferrite grain boundaries and N diffusion from supersaturated ferrite to austenite. The heat input and content of austenite and ferrite have important influences on the mechanical properties of duplex stainless steels [14,15,16,17]. Therefore, by changing the welding parameters, the welding heat input can be increased, the cooling rate of the weld can be reduced, and more time can be allowed for N diffusion and adsorption in the austenite phase, thus increasing the proportion of austenite in the HAZ and improving the mechanical properties of weld joints.

Compared to conventional TIG, P-TIG has a positive effect on the fluidity of the weld pool, as well as shorter residence time in the high temperature region, shorter grain growth time, and finer grain size in the weld metal zone. Arunkumar et al. [18] used P-TIG and TIG to join Al-Mg-Sc alloy and found that the microstructure size of TIG weld joints was larger than that of P-TIG joints, and the P-TIG process resulted in more loss of hardness compared to TIG. Junaid et al. [19] prepared a butt joint of 1.6 mm thick Ti-5Al-2.5Sn titanium alloy plate using pulsed laser beam welding (P-LBW) and P-TIG welding and found that the cooling rate of P-LBW was faster and the hardness in the fusion zone of P-LBW was higher than that of P-TIG weldments. The residual stress of P-LBW and P-TIG weldments showed a similar trend, but the smaller width of the heat affected zone of P-LBW resulted in a greater non-uniformity of stress distribution across the thickness.

Lower linear energy welding processes, such as plasma arc welding (PAW), laser beam welding (LBW), and EBW, have less austenite precipitation in the weld metal because of faster cooling rates, which leads to an imbalance in the ratio of the two phases in the weld joint [20,21,22,23]. Krasnorutskyi et al. [20] used EBW to weld thin plates of standard duplex stainless steel type 1.4462 and found that the cooling rate and chemical composition had crucial effects on the final results. At the same time, basic research on the influence of N escape and cooling rate was conducted, and the influence of the welding parameters on the formation of austenite and mechanical properties was considered, so as to develop an appropriate electron beam welding process technology. Despite the preheating of welded duplex stainless steel and use of electron beam multiple scanning to slow down the cooling rate and facilitate austenite precipitation, the austenite percentage in the weld zone was still less than 30 vol.%, which did not meet the requirements of a high-quality weld. In addition, electron beam energy is more concentrated, causing a certain amount of collapse, which worsened the quality of weld joint formation.

The macroscopic appearance of the weld joint has a great influence on the welding quality. Since the low heat input welding process cannot be avoided when welding thin-walled workpieces, it is possible to compensate for the loss of N by adding appropriate N_2_ into the shielding gas, promoting the precipitation of austenite, to balance the two-phase ratio of weld joints. Muthupandi et al. [24] used EBW and LBW methods to weld UNS 31803 duplex stainless steel, and respectively introduced Ni and N into the weld metal by adding N_2_ into the shielding gas and using high-Ni welding wire. It was found that the addition of Ni and N played an important role in the microstructure and impact toughness of weld joints. The addition of Ni or N contributed to a significant increase in the percentage of austenite in the weld joints of EBW and LBW. Gomez et al. [25] welded 2205 duplex stainless steel by using the pulsed GMAW process with ER2209 filler metal while changing the N_2_ content in the shielding gas from 0 to 6.4 vol.%. When the concentration of N_2_ in the shielding gas was too high, more spatter was caused during the welding process, and the arc became more unstable. No harmful phases, such as CrN, Cr_2_N, and σ, or other intermetallic compounds were detected in both the WZ and HAZ, confirming that the addition of N_2_ did not degrade the mechanical properties of weld joints. With the addition of 0–5 vol.% N_2_ in the shielding gas, the elongation of weld joints has been improved. Furthermore, researchers have diversified the use of advanced welding techniques, such as friction welding (FW), FSW, DW, active tungsten argon arc welding (A-TIG), hybrid welding, etc., by selecting appropriate filler metals and welding parameters, achieving high-quality joining of duplex stainless steels [26,27,28,29].

To sum up, P-TIG is suitable for butt jointing of thin-walled pipelines of UNS S32205 duplex stainless steel. Because of the pulse welding gap, the peak temperature between each welding point is guaranteed to be lower, and the stay time in the high temperature range is shorter, which effectively reduces the negative impact of heat accumulation during thin-walled pipeline welding on the formation of the weld joints and avoids problems such as weld pool collapse or penetration in weld joints [6,30,31,32]. At present, the research on duplex stainless steel weld joints is mainly focused on thick-walled plate butt joints. However, in the practical applications of chemical and marine engineering, there are often a large number of thin-walled pipelines, such as superheated pipelines in steam generators [33,34]. Within this context, this study makes a further contribution to the understanding of the fatigue performance of the joint of UNS S32205 duplex stainless steel thin tube.

Hence, the present study aims to simulate the fatigue service environment of joints through high cycle fatigue tests and study the stability of mechanical properties of weld joints under long-term service conditions and the sensitivity of fatigue life to the shielding gas N2 content in order to improve the fatigue life and corrosion resistance of duplex stainless steel welded pipes during service.

## 2. Materials and Methods

### 2.1. Experimental Materials

In this paper, 2205 duplex stainless steel pipe with a specification of Φ19 mm × 2 mm (outside diameter of 19 mm with a wall thickness of 2 mm) was taken to form the butt joint of a thin-walled pipeline. ER-2209 welding wire with a diameter of 1.2 mm was selected as the filler material. The chemical compositions of 2205 duplex stainless steel and ER-2209 welding wire are shown in Table 1.

### 2.2. Welding Parameters

The WSME-250D model P-TIG (HANKERELECTRiC, Shanghai, China) was used to weld 2205 duplex stainless steel pipes. The following welding parameters were used: base current *I*_b_ = 60 A, peak current *I*_p_ = 102 A, pulse frequency *f* = 10 Hz, pulse duty cycle *ψ* = 60%, and the welding speed was 2.5 mm/s. The N_2_ content of the shielding gas was set as 0 vol.% N_2_, 2 vol.% N_2_, 4 vol.% N_2_, and 6 vol.% N_2_, respectively, with a balance of Ar. Weld joints with different shielding gas N_2_ contents were obtained. The specific welding process parameters are shown in Table 2.

### 2.3. Welding Process

**Figure 1:**
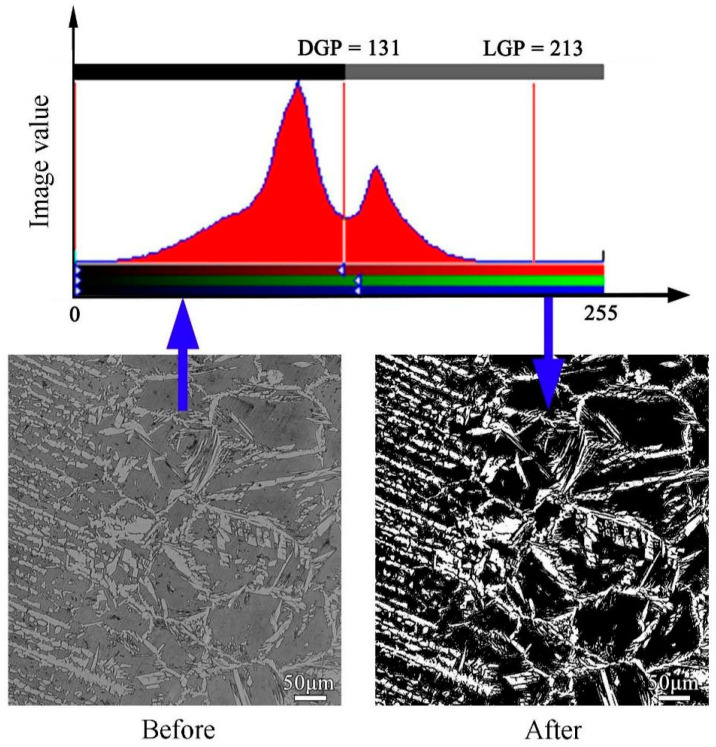
Metallographic image processing by IPP image software.

Metallographic specimens were prepared to detect the microstructure characteristics, including the two-phase ratio. The prepared specimens were etched with Beraha metallographic etching reagent (1 g potassium metabisulfite, 85 mL water, 15 mL HCl) for 15 s, followed by cleaning with alcohol and blowing dry. Image-Pro Plus 6.0 (IPP) image analysis software was used to calculate the two-phase ratio of metallographic micrographs. In this experiment, the calculation was carried out by taking the average of 3 area shots. The two-phase ratio detection process is shown in Figure 1. During the measurement process, the magnification of the metallographic diagram has a certain impact on the calculation results. When the magnification is too large, the microstructure is not representative, resulting in excessive randomness in the calculation results. When the magnification is too small, the grain distribution is relatively dense, the boundaries of the phase boundaries are unclear, and the image pixel recognition is not clear. So, we chose metallographic images with a magnification of 100 times for software processing. The grid intersection counting method uses ferrite as the counting benchmark. If the second intersection point is on the austenite phase, the count is 1, and for every 1% increase in intersection points, the count is 0.5 on the grain boundary between the ferrite phase and austenite phase. If the intersection point is on the ferrite phase, the count is 0. In order to reduce errors, this experiment selected three typical positions for each area of the weld joint and took the average value for calculation.

The MHVS-1000Z microhardness tester (LiDun, Shanghai, China) was used for testing the hardness of the different zones of the weld joint, with a loading load of 50 gf and unloading after 10 s. The hardness test of the weld joint was performed according to the ASTM A370-12a [35] standard.

**Figure 2:**
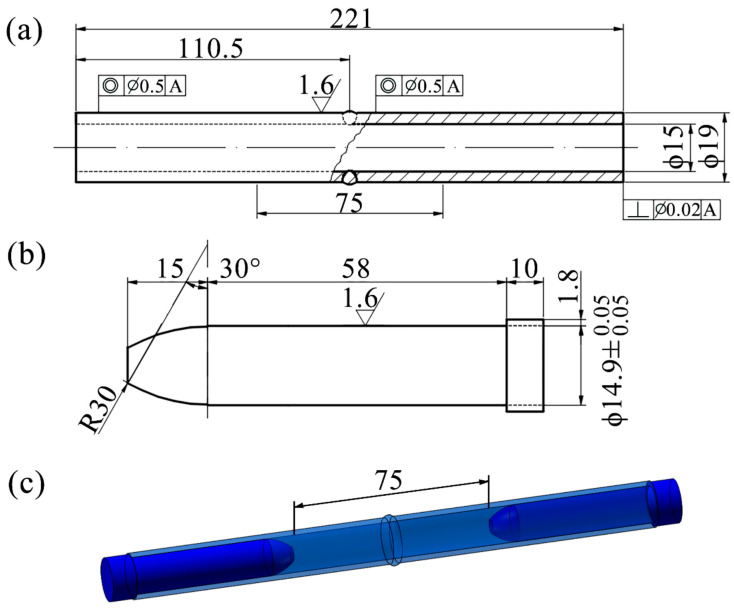
Thin-walled pipe butt joint specimen for mechanical property tests (unit: mm). (**a**) Specimen size; (**b**) Plug size; (**c**) Plug assembly drawing.

The tensile mechanical properties of the thin-walled pipe butt joint specimens were conducted by a SANS CMT4204 tensile testing machine (Shenzhen Chuyinghao Technology Co., Ltd., Shenzhen, China) at a rate of 0.5 mm/min. The specimens and filler plug dimensions were processed following the ASTM E8/E8M-13a [36] standard, as shown in Figure 2. The weld joints of each process parameter were tested three times, and the results were averaged.

The fatigue specimens were the same as the tensile specimens. The fatigue test adopted medium-voltage, high-frequency, constant-amplitude cyclic sine wave, the loading frequency was 10 Hz, and the stress ratio was *r* = 0.1. Three different stress amplitudes were used to test the weld joints under various process parameters. The maximum stress amplitudes were set as 0.6, 0.7, and 0.9 times the yield stress of the weld joints (in Table 3) and rounded, namely 250 MPa, 300 MPa, and 400 MPa, respectively. Tests on specimens at each stress amplitude were repeated 3 times.

## 3. Experimental Results and Analysis

### 3.1. Microstructure and Tensile Mechanical Properties

**Figure 3:**
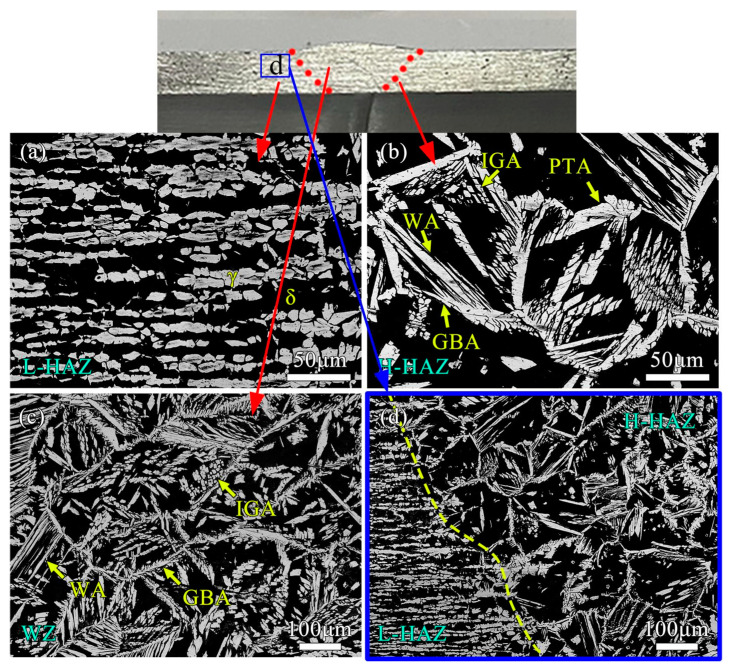
Microstructure of different regions of weld joints. (**a**) LT-HAZ; (**b**) HT-HAZ; (**c**) WZ; (**d**) HAZ.

The weld joint could be divided into four zones: base material (BM), low temperature heat-affected zone (L-HAZ), high temperature HAZ (H-HAZ), and weld zone (WZ). Figure 3a shows the metallographic structure of L-HAZ. The austenitic phase of LT-HAZ generally presented a lath shape, but some lath austenitic phases began to fracture. Figure 3b shows the metallographic structure of the H-HAZ. The austenite phase exhibited various forms, including grain boundary austenite (GBA), Widmanstä austenite (WA), intra granular austenite (IGA), and partially transformed austenite (PTA). The ferrite phase presented an irregular polygonal grid shape, with GBA surrounding the ferrite grain boundaries. The slender WA was distributed parallel to the ferrite phase, while the small IGA was randomly distributed. The average grain size of the ferrite was 70–100 μm.

Figure 3c shows the metallographic structure of the WZ. The GBA was thicker, with a large amount of WA arranged parallel to the ferrite grain boundaries, and a large amount of IGA filling the entire ferrite lattice. The average grain size of ferrite was 200–300 μm. By comparing Figure 3b,c, it can be seen that due to the lower welding heat input and peak temperature received by H-HAZ compared to WZ, the residence time in the high-temperature stage was shorter, and the time for ferrite growth and austenite precipitation was also shorter. Therefore, the grain size of ferrite in H-HAZ was smaller, and the proportion of austenite was much smaller than that of WZ. On the left side of Figure 3d is L-HAZ, and on the right side is H-HAZ. It can be seen that the size of ferrite continued to increase and the proportion of austenite also increased as it approached the center of the weld.

**Figure 4:**
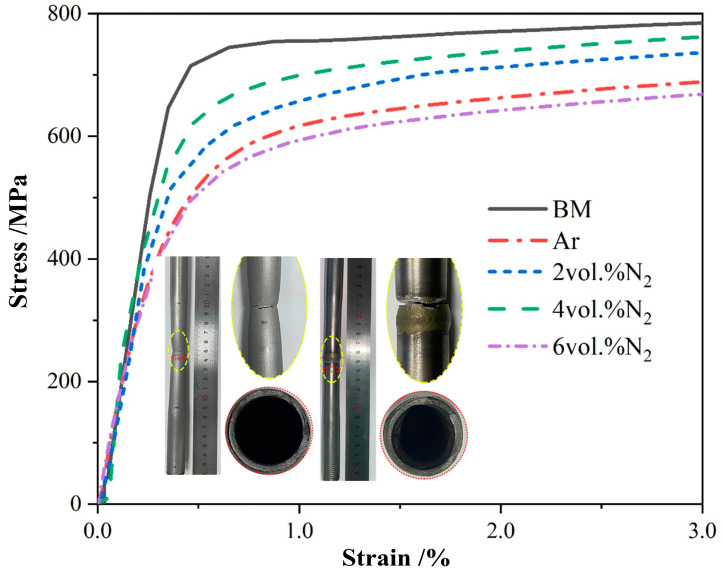
Stress–strain curve of weld joints with different N_2_ contents.

The tensile mechanical properties of BM and the thin-walled pipe butt joints with different shielding gas N_2_ contents are shown in Figure 4 and Table 3. The degradation of mechanical properties was obvious in the weld joint compared to that of BM. The yield stress (*Rp*_0.2_), tensile stress (*R_m_*), and ductility (*A*) were all decreased compared with those in BM. However, for the weld joints with 2 vol.% N_2_ and 4 vol.% N_2_, the mechanical properties and ductility were very close to those of BM. Thus, increasing the N_2_ content in the Ar shielding gas could increase the strength and ductility, especially for the weld joint with 4 vol.% N_2_.

### 3.2. Fatigue Performance Analysis of Pipe Weld Joints

**Figure 5:**
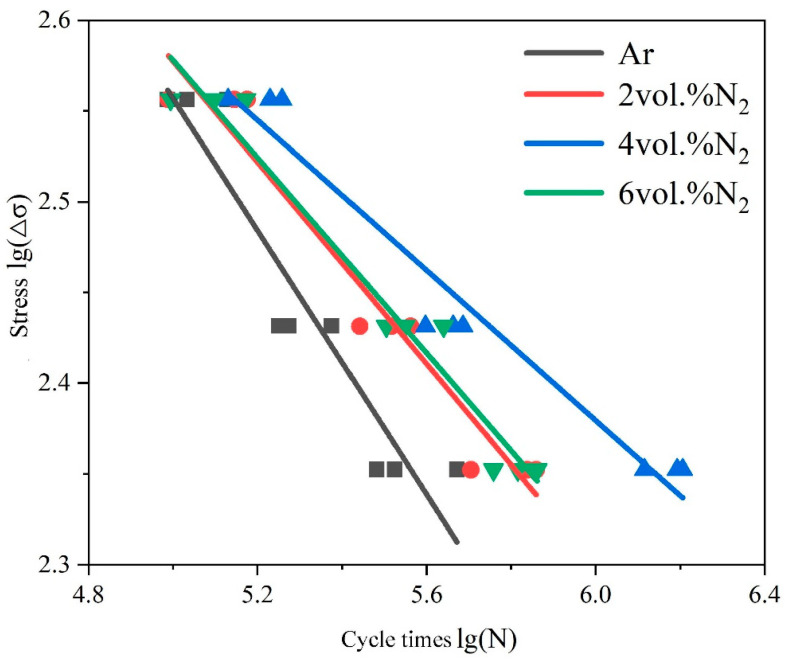
S-N curves of different weld joint specimens.

Fatigue tests were conducted for the weld joints of thin-walled pipe under different shielding gases, and the results in the fatigue S-N curves [37,38] are shown in Figure 5.

It can be clearly seen that the change in the S-N curve of the weld joint under Ar-4 vol.% N_2_ shielding gas occurred more gently, indicating that the fatigue strength in the long-life zone was significantly higher than the other parameters after 10^6^ cycles. The fatigue strengths of the weld joints under Ar-2 vol.% N_2_ and Ar-2 vol.% N_2_ shielding gases were second only to that of the weld joint under Ar-4 vol.% N_2_. The fatigue strength of the weld joint under Ar shielding gas was the worst. The obtained results in this figure indicated that the scatter band in the fatigue lifetime became wider at 105.6 to 106 cycles. The corresponding median fatigue strengths of the weld joints under different shielding gases at 2 × 10^6^ cycles were 121.3 MPa, 164.3 MPa, 207.5 MPa, and 169.0 MPa, respectively. It can be seen that the fatigue performance first increased and then decreased with the increase in N_2_ content in the shielding gas, and the fatigue performance was the strongest under Ar-4 vol.% N_2_ shielding gas. The fractured initiation sources of the fatigue specimens were all in the high temperature heat-affected zone (H-HAZ) of the weld joints, which was determined by the shape of the fusion welding seam, as shown in Figure 4. The weld joints all had a certain welding excess height, resulting in stress concentration at the H-HAZ position. The two-phase ratio of H-HAZ was seriously unbalanced, which reduced the connection quality in this zone, as shown in Figure 1 and Figure 3. The ferrite grain size of H-HAZ was refined in the weld joints under the addition of N_2_ in the shielding gas compared with Ar shielding gas. Under fatigue load, the coarse grains were more likely to initiate fatigue cracks, thereby reducing the fatigue life of weld joints [39,40,41,42].

### 3.3. Effect of Different Stress Levels on Fatigue Fracture

**Figure 6:**
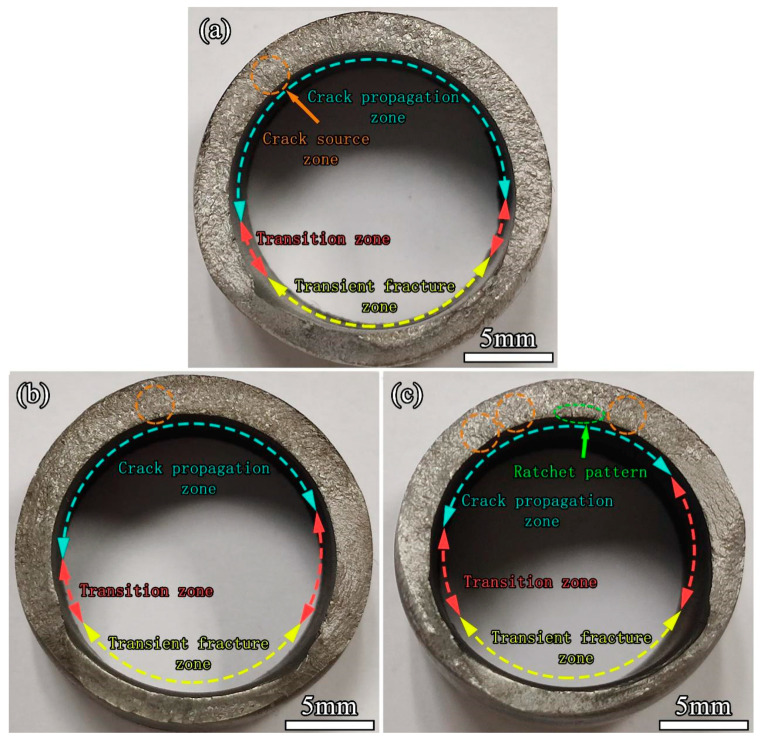
Macroscopic morphology of fractures at different stress levels: (**a**) 225 MPa; (**b**) 270 MPa; (**c**) 360 MPa.

In the actual service environment of the project, the pipe may face the influence of different cyclic loads [43,44,45]. In order to study the influence of the stress level on the fatigue performance of the thin-walled P-TIG butt joint of the duplex stainless steel more widely, this part will discuss the weld joint with Ar-4 vol.% N_2_ and fatigue tests were carried out at stress levels of 225 MPa, 270 MPa, and 360 MPa, respectively. Figure 6 shows the macroscopic topography of fatigue fractures obtained at different stress levels. It can be seen from the figure that the fatigue fractures under different stress levels were mainly divided into three typical zones [46,47]: the fatigue crack source zone, the fatigue crack propagation zone, and the transient fracture zone. It can be also observed that as the stress level increased, the proportion of the crack propagation zone in the fatigue fracture to the total fracture area decreased. There were obvious “step-like” [47,48] lines in the transition zone between the crack propagation zone and the transient fracture zone, which was caused by rapid crack propagation caused by the alternating load after the crack expanded to a certain size. It can be seen that the area of the transition zone increased with the increase in the stress level. The thickness of the pipe wall in the transient fracture zone was obviously smaller than that in the crack propagation zone.

**Figure 7:**
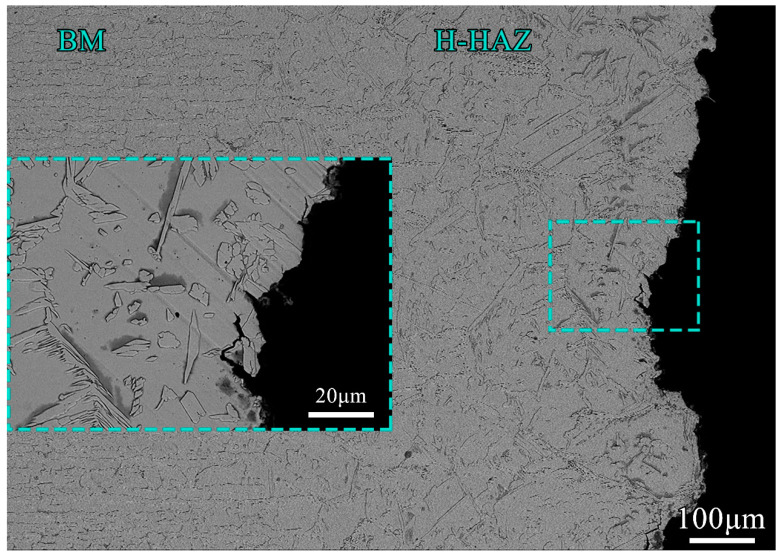
Fatigue fracture section morphology of weld joint.

The transient fracture zone was caused by rapid fracture because the strength of the weld joint was not enough to support the tensile load applied at both ends after the fatigue crack expanded to a certain extent. Therefore, the appearance of the transient fracture zone was similar to that of the tensile fracture. The shear lip zone could be seen on both sides of the pipe wall because the duplex stainless steel had good ductility [49,50,51]. There was an obvious ratchet pattern on the fracture at the stress level of 360 MPa [52,53] because the fracture form of the weld joint was a multi-source fatigue fracture. Under the stress levels of 225 MPa and 270 MPa, fatigue stress concentration did not easily occur at the weld joint, and the source of fatigue crack initiation was singular. Meanwhile at the high stress level, there were multiple sources of fatigue crack initiation. In order to further observe the cross-section of the fatigue fracture, the weld joint of the pipe was axially cut at the crack source area. An SEM image of the fatigue fracture section of a weld joint is shown in Figure 7. It can be seen that the fatigue fracture occurred at the H-HAZ of the weld joint, and there was a relatively obvious morphology of the grid-like ferrite phase. The fractured form was mainly a transgranular fracture.

**Figure 8:**
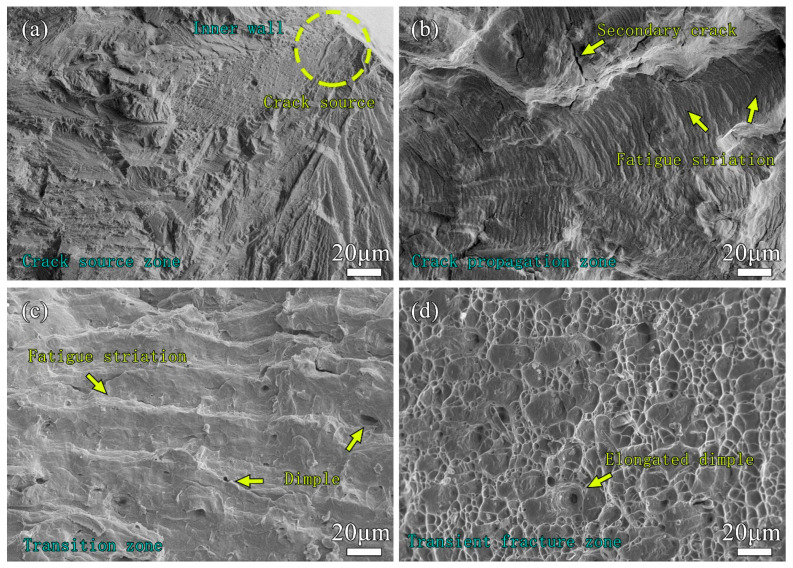
SEM images of fatigue fracture zone: (**a**) crack initiation region; (**b**) propagation area; (**c**) transition region; (**d**) transient breaking.

The morphology of each typical area of the fatigue fracture is shown in Figure 8. The effect of the defects on the surface of the fatigue sample was equivalent to that of a sharp notch, so it very easily caused stress concentration and led to the initiation of fatigue cracks, such as the pits on the inner surface of the pipe wall shown in Figure 8a. This defect was caused by the action of local shear stress. Dislocation migration will occur on the surface of the material under the action of maximum shear stress, and dislocation migration will promote the formation of fine slip bands [54]. The number of slip bands is positively related to the fatigue stress, and the greater the fatigue stress, the more slip bands. When the specimen is subjected to repeated fatigue loads, reverse slip will occur on the adjacent slip surfaces. With increasing numbers of slip bands, grooves and ridges on the material surface will continue to form, which will eventually lead to the initiation of fatigue cracks [55,56]. The propagation of fatigue cracks can be mainly divided into two stages. In the first stage, the fatigue crack propagates inwards along the slip plane infinitely approaching 45 degrees to the principal stress. At this stage, it is difficult to effectively observe the changes in the microscopic section because the amount of crack propagation in each cycle is very small. In addition, the fracture at this stage is similar to the cleavage morphology, and no plastic deformation features are found. In the second stage, the crack propagates over a period of time until it encounters obstacles, such as grain boundaries or inclusions.

The crack needs to overcome the obstacle of the grain and cross the grain boundary. The action direction of the principal stress at the crack tip will deviate after the crack grows to a certain length and gradually turns to the direction perpendicular to the direction of the maximum normal stress. In this stage, the transgranular crack grows at a faster rate, and the further away from the fatigue source, the faster the growth rate. The main feature of the fracture at this stage is the appearance of fatigue striations, as shown in Figure 8b. The transition zone in Figure 8c was located at the junction of the fatigue crack propagation zone and the transient fracture zone, where fatigue striations and dimples appeared at the same time, and the distance between fatigue striations was relatively large, about 10 μm. When the stress intensity factor at the crack tip exceeds the critical stress intensity factor, the crack becomes unstable and expands rapidly, and the weld joint of the pipe is rapidly pulled apart. The fracture morphology shown in Figure 8d was consistent with the tensile fracture of the weld joint of the pipe, with elongated dimples appearing.

### 3.4. Effect of Shielding Gas Nitriding on Fatigue Fracture

Under the continuous action of alternating load, the fatigue life behavior is related to the change in the internal microstructure of weld joints [57,58,59]. Different N_2_ contents of the shielding gas will affect the distribution of the two-phase structure and also change the grain size of the weld joint, which has a crucial impact on the fatigue performance of the thin-walled pipe butt joint [60,61]. At the high stress level, the effect of the N_2_ content of the shielding gas on weld joints was not as obvious as that at the low stress level. Higher stress levels will cause fatigue cracks to initiate rapidly, resulting in insignificant effects of shielding gas nitriding on the fatigue life. It can be observed from Figure 5 that the fatigue cycle times of weld joints with different N_2_ contents of the shielding gas under the high stress level were relatively close, while the fatigue cycle times of weld joints under the stress level of 225 MPa were significantly different. Therefore, this part focuses on the effect of different N_2_ contents of the shielding gas on the fatigue fracture of pipe weld joints under the stress level of 225 MPa.

The area of the crack propagation zone of the weld joints without N_2_ and with excessive N_2_ in the shielding gas was small, and the area of the transition zone and transient fracture zone was large. The fatigue life of the weld joint under Ar-4 vol.% N_2_ shielding gas was longer, and the number of load cycles experienced was more than the others, so the area of the crack propagation zone was the highest.

**Figure 9:**
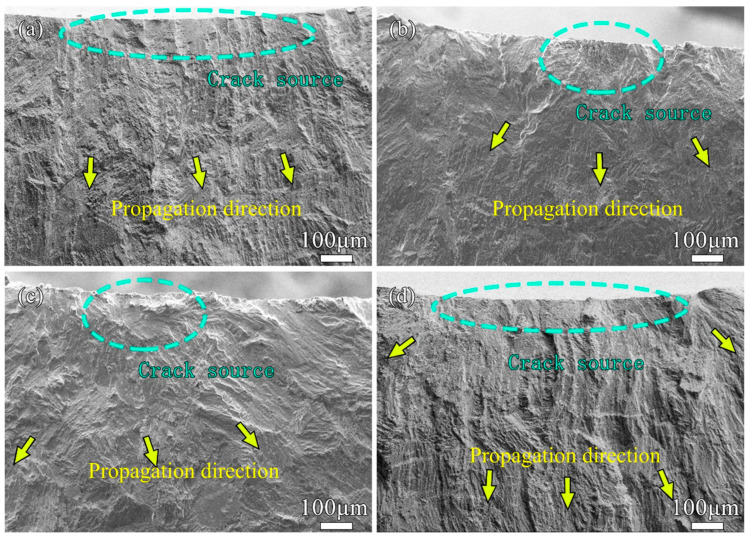
Morphology of crack source region under different N_2_ contents: (**a**) Ar; (**b**) 2 vol.%; (**c**) 4 vol.%; (**d**) 6 vol.%.

SEM images of the crack source zones of the fatigue fractures of pipe weld joints are shown in Figure 9. By magnifying the crack source zone of the fatigue fracture under different N_2_ contents, it can be clearly seen that the tear ridge exhibited directionality and the direction of the arrows is the direction of crack propagation. In Figure 9b,c, the tear ridges expanded around the crack source zone, the fatigue crack growth zone was distributed radially, the uplift of the tear ridge was not obvious, and the crack growth zone was relatively flat. In Figure 9a,d, the crack tended to expand towards the outer wall of the pipe weld joint and the uplift of the tear ridge was more obvious, which showed that the crack source of the pipe weld joints under Ar and Ar-6 vol.% N_2_ shielding gases was not limited to a small area, but multiple crack sources appeared in a large area on the inner wall surface of the pipe. Therefore, the tear ridges in the fatigue crack growth zone extended to the outer wall in a more parallel manner. This was related to the microstructure of the H-HAZ of the weld joint. Under shielding gases of Ar-2 vol.% N_2_ and Ar-4 vol.% N_2_, the two-phase ratio of H-HAZ was relatively balanced, and crack initiation usually occurred from a single defect under a low stress load cycle. However, the contents of the ferrite phase in the H-HAZ under Ar and Ar-6 vol.% N_2_ shielding gases were high, which destroyed the original two-phase balance of duplex stainless steel and seriously affected the fatigue performance of weld joints.

**Figure 10:**
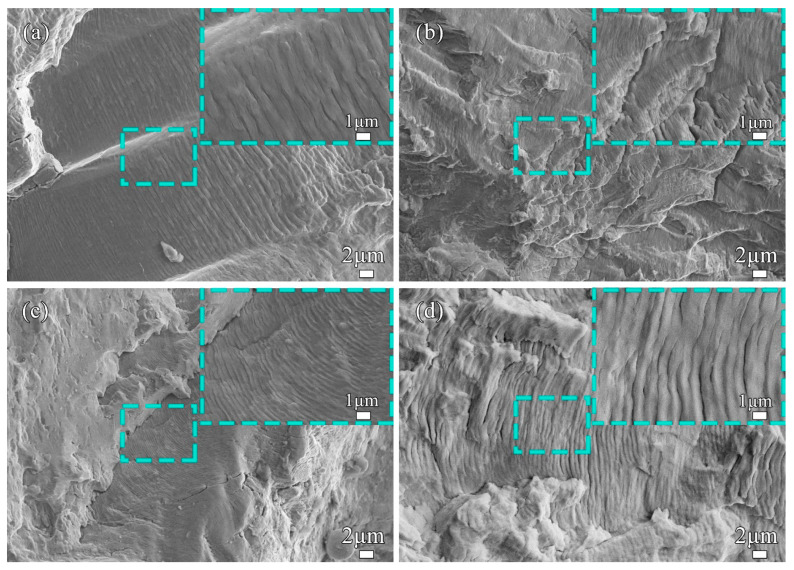
Morphology of crack propagation zone under different N_2_ contents: (**a**) Ar; (**b**) 2 vol.%; (**c**) 4 vol.%; (**d**) 6 vol.%.

SEM was performed on the crack propagation zone near the above-mentioned crack source zone, as shown in Figure 10. It can be seen that dense fatigue striations were distributed in a wave shape, and the direction of these fatigue striations was perpendicular to the local propagation direction of the fatigue crack. Fatigue striations refer to the microscopic marks left on the fracture surface when metal materials undergo fatigue fracture under the action of cyclic alternating loads [62]. The fatigue striations will continue to expand on different planes, which may not be continuous, but the fatigue striations on each plane are continuous and parallel. When the fatigue striations on different planes gather, they show bright edges, as shown in Figure 10b. From the morphology of the fatigue striations, it can be seen that the fatigue striations at the crack propagation stage of weld joints under Ar and Ar-6 vol.% N_2_ shielding gases were relatively clear and continuous, and the indentations of the fatigue striations were deeper and the spacing was larger, which indicated that the crack tip had a longer propagation distance after an alternating load cycle. Therefore, the fatigue lives of the two weld joints under Ar and Ar-6 vol.% N_2_ shielding gases were shorter. Under the same stress level, the distance between the fatigue striations of the weld joint under Ar-4 vol.% N_2_ was the smallest, as shown in Figure 10c, and its fatigue life was the longest compared with other weld joints. By SEM, the average width of the fatigue striations was counted in different planes. Multiple planes were measured on the crack propagation zone near the crack source zone for each weld joint. The statistical data are shown in Table 4. It is clearly seen that the average distances of the fatigue striations of the weld joints under Ar-2 vol.% N_2_ and Ar-4 vol.% N_2_ were distributed in a narrow range with small values, meaning that these two kinds of weld joints possessed better fatigue properties.

This might due to the fact that shielding gas nitriding effectively improved the balance of the two-phase ratio and the hardness of the austenite phase, optimized the internal slip system, inhibited the proliferation of dislocations in the austenite phase, and improved the fatigue life of weld joints.

As mentioned above, shielding gas nitriding effectively improved the fatigue life of weld joints. However, this research only focused on the thin-walled pipe butt joint formed by P-TIG. There would be many factors that may influence the effect of nitriding, such as the material (2304, 2507), welding method (laser welding, gas metal arc welding), weld plate size, and welding parameters. Therefore, shielding gas nitriding can be further studied, including the influences of nitriding on the microstructure and mechanical properties under the above factors. Moreover, thin-walled duplex stainless steel pipes are widely used in heat exchangers and in the field of deep-sea transportation; industrial applications also need be further investigated.

## 4. Conclusions

1. Fatigue tests at three stress levels of 225 MPa, 270 MPa, and 360 MPa were carried out on thin-walled pipe butt joints with different N_2_ contents of the shielding gas, and the fatigue specimens were all fractured at the H-HAZ. The median fatigue strength of the weld joint under Ar-4 vol.% N_2_ was 207.5 MPa, which was significantly higher than that under other shielding gases.
2. For the thin-walled pipe butt joint, the area proportion of the crack propagation zone continuously increased with increasing fatigue stress level. At the 360 MPa stress level, the fatigue crack sources exhibited multiple origins. As the stress level decreased to 270 MPa and 225 MPa, the area proportion of the crack extension area under Ar-4 vol.% N_2_ was the highest, and fatigue crack source of fatigue crack initiation was singular.
3. By using shielding gas nitriding, the high cycle fatigue property of the butt weld joint of thin-walled pipe can be obviously improved. Austenite and ferrite phase ratio balance was achieved for the microstructure of the weld joint, and the strength and ductility were also increased, especially for the weld joint under Ar-4 vol.% N_2_ shielding gas. Additionally, the yield strength of weld joints was as high as 570 MPa, and the elongation rate was as high as 32.3%. Therefore, shielding gas nitriding can effectively improve the fatigue properties of thin-walled pipe butt joints.

## Figures and Tables

**Table 1:** Chemical compositions of duplex stainless steel and welding wire.

Type	C	Cr	Ni	Mo	N	Other	Fe
BM	0.022	22.74	4.92	2.84	0.16	1.689	Bal.
ER-2209	0.018	23.04	8.6	3.00	0.16	1.42	Bal.

**Table 2:** P-TIG welding parameters.

Number	Shielding Gas	Welding Speed (cm/min)	Peak Current (A)	Welding Voltage (V)
1	Ar	150	102	22
2	2 vol.% N_2_	150	102	22
3	4 vol.% N_2_	150	102	22
4	6 vol.% N_2_	150	102	22

**Table 3:** Tensile mechanical properties of weld joints.

	*R_m_*/MPa	*R_P_*_0.2_/MPa	*A*/%	Fracture Position
BM	811	655	32.8	BM
Ar	768	465	17.5	HAZ
2 vol.% N_2_	784	540	30.7	WZ
4 vol.% N_2_	798	570	32.3	BM
6 vol.% N_2_	776	445	20.4	WZ

**Table 4:** Statistical data for the width of fatigue striations in different fatigue crack propagation zones of the weld joints.

	Width Range/μm	Number of Planes	Minimum Number on One Plane
Ar	0.52–1.24	8	20
2 vol.% N_2_	0.23–0.86	8	30
4 vol.% N_2_	0.28–0.79	8	30
6 vol.% N_2_	0.57–1.32	8	20

## Data Availability

The raw/processed data will be made available upon request.
